# Phytochemical investigation and antimicrobial activity of *Psidium guajava* L. leaves

**DOI:** 10.4103/0973-1296.66939

**Published:** 2010

**Authors:** A. M. Metwally, A. A. Omar, F. M. Harraz, S. M. El Sohafy

**Affiliations:** *Department of Pharmacognosy, Faculty of Pharmacy, Alexandria University, Alexandria, Egypt*

**Keywords:** Antimicrobial activity, guava leaves, *Psidium guajava* L, quercetin glycosides, quercetin, quercetin-3-O-β-D-arabinopyranoside

## Abstract

*Psidium guajava* L. leaves were subjected to extraction, fractionation and isolation of the flavonoidal compounds. Five flavonoidal compounds were isolated which are quercetin, quercetin-3-O-α-L-arabinofuranoside, quercetin-3-O-β-D-arabinopyranoside, quercetin-3-O-β-D-glucoside and quercetin-3-O-β-D-galactoside. Quercetin-3-O-β-D-arabinopyranoside was isolated for the first time from the leaves. Fractions together with the isolates were tested for their antimicrobial activity. The antimicrobial studies showed good activities for the extracts and the isolated compounds.

## INTRODUCTION

*Psidium guajava* L. leaf (family Myrtaceae) has a long history of folk medicinal uses in Egypt and worldwide as a cough sedative, an anti-diarrheic, in the management of hypertension, obesity and in the control of diabetes mellitus.[1–7] The leaf extract was found to possess anticestodal,[8] analgesic, anti-inflammatory properties,[9] antimicrobial[10] hepatoprotective[11] and antioxidant activities.[12] In addition, the leaf extract is used in many pharmaceutical preparations as a cough sedative.

Guava leaf extract contains flavonoids, mainly quercetin derivatives, which are hydrolyzed in the body to give the aglycone quercetin which is responsible for the spasmolytic activity of the leaves.[4] Quercetin has several pharmacologic actions; it inhibits the intestinal movement, reduces capillary permeability in the abdominal cavity[13] and possesses dose-dependent antioxidant properties,[14] anti-inflammatory activity,[15–21] antiviral and antitumor activities.[22–27] It also inhibits the aldose reductase enzyme.[28] It should be noticed that most of the flavonoidal constituents of guava leaf are quercetin derivatives, namely, quercetin, avicularin, guaijaverin, isoquercetin, hyperin, quercitrin, quercetin 3-O-gentiobioside, quercetin 4’-glucuronoide.[4,29–33]

## MATERIALS AND METHODS

### Plant material

P. *guajava* L. leaf was collected from El tahrir (Alexandria-Cairo desert road) during spring while the fruits are premature. A specimen is deposited in the department of Pharmacognosy, Faculty of Pharmacy, Alexandria University, Egypt.

### Reference materials

Quercetin, glucose, galactose, L-arabinose and d-arabinose were supplied by E. Merck (Darmstadt, Germany). Quercetin-3-β-D-glucoside and quercetin-3-β-D-galactoside were supplied by Sigma-Aldrich Chemie GmbH (Steinheim, Germany).

### Solvents

Petroleum ether (40-60°C), chloroform, ethyl acetate, *n*-butanol, methanol and ethanol were of analytical grade.

### Chromatographic requirements

Precoated thin layer chromatography (TLC) plates (silica gel 60F-254) with the adsorbent layer thickness of 0.25 mm (E-Merck), silica gel (Merck) and kieselgel 60, 0.063-0.20 mm for column chromatography (E-Merck) were used.

### Special apparatus

Melting points were determined using Sturat SMP heating stage microscope and were uncorrected. UV spectra were obtained on Pye Unicam SP8-100 UV/VIS spectrophotometer and Perkin-Elmer, Lambada 3B UV/VIS spectrophotometer. Nuclear magnetic resonance (NMR) analyses were recorded on JOEL 500 MHz and Bruker Avance 300 MHz spectrometers. Mass spectral analyses were recorded on VG 7070 E-HF.

### Extraction, fractionation and isolation

The air-dried powdered *P. guajava* leaves (1 kg) were exhaustively extracted with 50% ethanol at room temperature. The extract was filtered and concentrated under reduced pressure at 60°C to about 0.5 l and then successively fractionated with petroleum ether, chloroform, ethyl acetate, and *n*-butanol. Each extract, as well as the interface formed between the chloroform and aqueous layer, were separately concentrated and freed from solvent. Six fractions were obtained: petroleum ether (0.6 g), chloroform (2.7 g), interface formed between chloroform and aqueous phase (10 g), ethyl acetate (9.7 g), *n*-butanol (22.8 g) and the remaining aqueous extract (9.5 g).

A portion of the ethyl acetate extract (3.5 g) was chromatographed on a 150-g silica gel column (3.5 cm diameter × 30 cm length).

Elution was started with chloroform:ethyl acetate mixture (8:2). Then, the polarity was increased using methanol gradually. Thirty-one fractions of 250 ml in each were collected, screened chromatographically using solvent system chloroform-ethyl acetate-methanol in the ratios (8:2:1) and (8:2:2).

### Isolation of material “ A ”

Fractions 12-15 containing 4-6% methanol showed a major spot of R_f_ 0.46 [chloroform-ethyl acetate-methanol (8:2:1)] that gave a yellow color with ammonia. It was purified from the other minor spots by repeated crystallization from methanol, yielding yellow crystalline needles (21 mg). R_f_ 0.86 [ethyl acetate-methanol-water-acetic acid (100:2:1:4 drops)], m.p. 316-318°C, soluble in methanol, acetone, dilute alkali and gives a canary yellow color with AlCl_3_. The UV spectral data, γ_max_ nm, are illustrated in Table 1; [M]^+^ *m/z* 302. ^1^H-NMR spectral data (300 MHz, CD_3_ COCD_3_ ) and ^13^C-NMR spectral data (75 MHz, CD_3_ COCD_3_ ) are shown in Table 2.

**Table 1:** UV spectral data of the compounds “A”, “B” and “C”

Spectrum	λ_max_ (nm)						
	Flavonoid “A”		Flavonoid “B”		Flavonoid “C”	
	Band I	Band II	Band I	Band II	Band I	Band II
MeOH	371	253	354.5	256	358	256
MeOH + NaOMe	398.5, 318 sh	271.5	408	272	411.5	272
MeOH + AlCl_3_	449.2	269	432	274	437	273.5
MeOH + AlCl_3_+HCl	421.5, 358.1 sh	266.9	402, 363.5 sh	270.5	405, 365 sh	268.5
MeOH + NaOAc	398.4, 323.4 sh	269.2	407	270	412	272

sh: shoulder

**Table 2:** NMR data of flavonoids A, B and C

	Flavonoid “A”		Flavonoid “B”		Flavonoid “C”	
	δH (ppm)	δC (ppm)	δH (ppm)	δC (ppm)	δH (ppm)	δC (ppm)
2	—	147.2	—	157.7	—	157.4
3	—	137.2	—	133.3	—	134.3
4	—	177	—	178.4	—	178.2
5	—	162.7	—	161.5	—	161.7
6	6.13, d, *J* = 2.1 Hz	99.5	6.21, d, *J* = 2.1 Hz	98.3	6.21, d, *J* = 2.1 Hz	100.3
7	—	165.3	—	164.5	—	164.8
8	6.4, d, *J* = 2.1 Hz	94.8	6.41, d, *J* = 2.1 Hz	93.2	6.41, d, *J* = 2.1 Hz	95.1
9	—	158.2	—	156.9	—	157
10	—	104.5	—	104	—	104.3
1‘	—	124.1	—	121.2	—	121
2‘	7.71, d, *J* = 2.15 Hz	116.2	7.53, d, *J* = 2.1 Hz	115.2	7.76, d, *J* = 2.2 Hz	116.2
3‘	—	146.2	—	144.8	—	144.7
4‘	—	148.7	—	148.2	—	148.7
5‘	6.86, d, *J* = 8.5 Hz	116.6	6.92, d, *J* = 8.3 Hz	114.8	6.88, d, *J* = 8.5 Hz	114.9
6‘	7.57, dd, *J* = 2.15, 8.5 Hz	121.8	7.51, dd, *J* = 2.1, 8.3 Hz	121.4	7.58, dd, *J* = 2.2, 8.5 Hz	121.7
1“	—	—	5.48, br s	107.9	5.17, d, *J* = 6.5 Hz	105
2“	—	—		81.7		73.3
3“	—	—	3.85-3.94	77.1	3.81-3.941	74.5
4“	—	—		86.4		69.5
5“	—	—		60.9		67.4

### Isolation of material “B”

Fractions 18-20 containing 12-15% methanol showed a major spot of R_f_ 0.57 [chloroform-ethyl acetate-methanol (8:2:2)] that gave a yellow color with ammonia. It was purified by repeated crystallization (12 mg). R_f_ 0.55 [ethyl acetate-methanol-water-acetic acid (100:2:1:4 drops)], m.p. 209-211°C, soluble in methanol, acetone, dilute alkali and gives a canary yellow color with AlCl_3_, gives positive molisch’s test. The UV spectral data, γ_max_ nm, are illustrated in Table 1.^1^ H-NMR spectral data (300 MHz CD_3_ OH) and ^13^ C-NMR spectral data (75 MHz, CD_3_ OH) are shown in Table 2. Long range ^1^H-^13^C correlation data as determined by HMBC experiments of flavonoid “B” are shown in Table 3.

**Table 3:** Long range ^1^H-^13^C correlation data as determined by HMBC experiments of flavonoid “B”

Carbon number	HMBC		
	Flavonoid B		
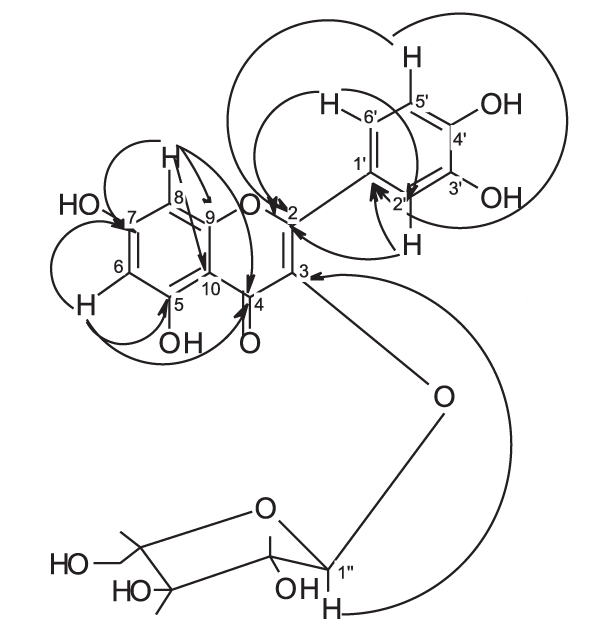			
2	6.92 (H-5’)	7.51(H-6’)	7.53(H-2’)
3	5.48(H-1")	—	—
4	6.21(H-6)	6.41(H-8)	—
5	6.21(H-6)	—	—
7	6.21(H-6)	6.41(H-8)	—
9	6.41(H-8)	—	—
10	6.41(H-8)	—	—
1’	7.53(H-2’)	6.92(H-5’)	—
2’	7.51(H-6’)	6.92(H-5’)	—
3’	7.51(H-6’)	7.53(H-2’)	6.92(H-5’)
4’	7.51(H-6’)	7.53(H-2’)	6.92(H-5’)
6’	7.53(H-2’)	6.92(H-5’)	—

### Isolation of materials “C”, “D” and “E”

Fractions 23-29 (0.3 g) containing 17.5-20% methanol showed four spots (giving a yellow color with ammonia) of R_f_ values 0.61, 0.49, 0.4, 0.35 [ethyl acetate-formic acid-acetic acid-water (25:2:2:4)]. It was rechromatographed on 60 g silica gel column (2.5 cm diameter × 30 cm length). The column was eluted with chloroform, with increasing concentrations of ethyl acetate (0-50%) and then increasing concentrations of methanol. Fractions 13 (chloroform-ethyl acetate (1:1) containing 4% methanol) was crystallized to give 8 mg. Meanwhile, fraction containing 6% methanol in chloroform-ethyl acetate (1:1) was purified by crystallization to give compound “C” (19 mg).

Material “C”: R_f_ 0.41 [ethyl acetate-methanol-water-acetic acid (100:2:1:4 drops)], m.p. 264-267C, soluble in methanol, acetone, dilute alkali and gives a canary yellow color with AlCl_3_, gives positive molisch’s test. The UV spectral data, λ_max_ nm, are illustrated in Table 1. ^1^H-NMR spectral data (300 MHz CD_3_ OH) and ^13^C-NMR spectral data (75 MHz, CD_3_ OH) are shown in Table 2.

Crystallization of fraction containing 10% methanol in chloroform-ethyl acetate (1:1) yielded a mixture of two compounds which were separated by preparative TLC using ethyl acetate-formic acid-acetic acid-water (25:2:2:4) with double run to give compound “D“ (10 mg) and compound “E” (11 mg).

Material “D”: R_f_ 0.31 [ethyl acetate-methanol-water-acetic acid (100:2:1:4 drops)], m.p. 240-243°C, soluble in methanol, acetone, dilute alkali and gives a canary yellow color with AlCl_3_, gives positive molisch’s test.

Material “E”: R_f_ 0.24 [ethyl acetate-methanol-water-acetic acid (100:2:1:4 drops)], m.p. 237-239°C, soluble in methanol, acetone, dilute alkali and gives a canary yellow color with AlCl_3_, gives positive molisch’s test.

### Acid hydrolysis of flavonoids “B”, “C”, “D” and “E”

Three milligrams of each compound was separately dissolved in a mixture of 0.5 ml methanol and 1 ml 2N hydrochloric acid. The solutions were then heated under reflux for 2 h, cooled, diluted with 1 ml water and the aglycones were extracted with ethyl acetate. The aglycones of “B”, “C”, “D” and “E” were identified to be quercetin by co-chromatography using reference compounds and chromatographic system chloroform-ethyl acetate-methanol (8:2:1). The aqueous solutions were neutralized with 5% Na_2_ CO_3_ solution and concentrated. The sugar moieties of “B”, “C”, “D” and “E” were identified by TLC in comparison with authentic reference materials, using chloroform-methanol (6:4) and visualized by methanol/H_2_ SO_4_ spray reagent. The structures of the isolated compounds are given in Table 4.

**Table 4:** The structures of the isolated compounds

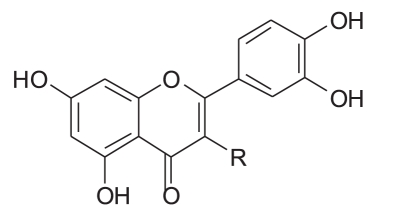		
Isolated compound	R	Notes
Quercetin	-OH	
Quercetin-3-O-α-l-arabinofuranoside	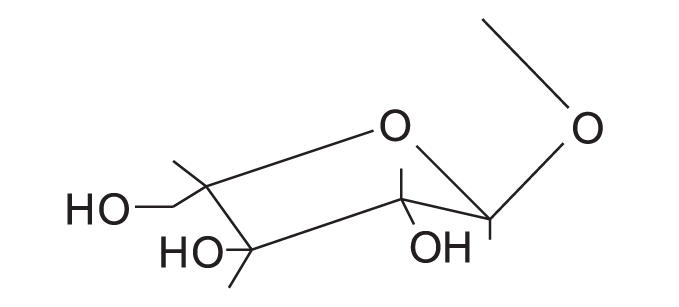	Avicularin
Quercetin-3-O-β-D-arabinopyranoside	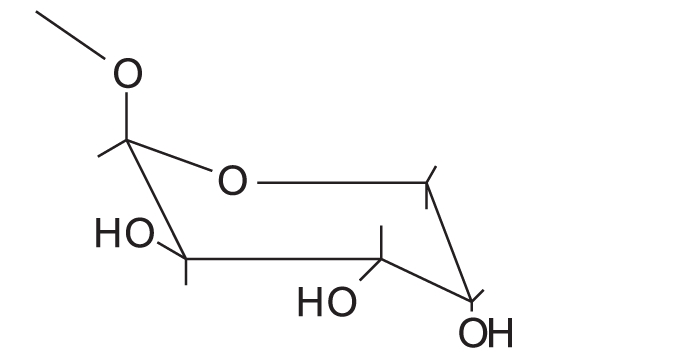	First report of its isolation from guava leaves
Quercetin 3-O-β-D-glucoside	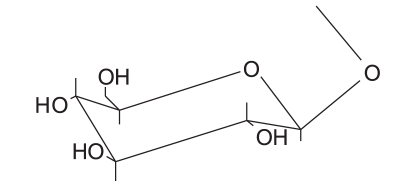	Isoquercetin
Quercetin 3-O-β-D-galactoside	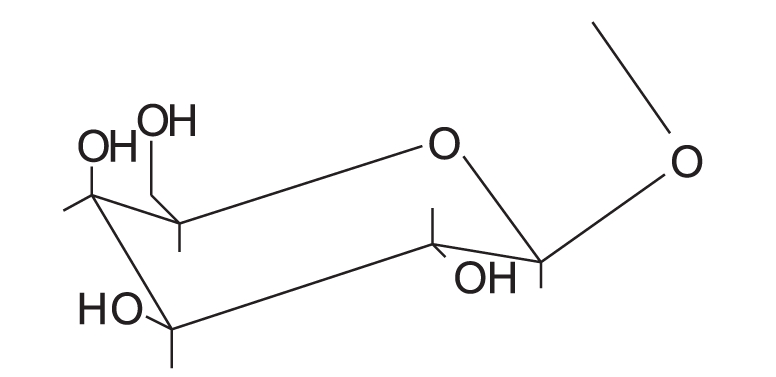	Hyperin

### Antimicrobial activity

Antibacterial and antifungal activities were determined using the agar diffusion technique[34] against the gram-positive bacterium *Staphylococcus aureus (S. aureus)*, two gram-negative bacteria, *Escherichia coli (E. coli)* and* Pseudomonas aeruginosa (P. aeruginosa),* and the fungus *Candida albicans (C. albicans).* The used organisms are local isolates provided from the Department of Microbiology, Faculty of Pharmacy, University of Alexandria.

One milliliter of 24-h broth culture of each of the tested organisms was separately inoculated into 100 ml of sterile molten nutrient agar maintained at 45°C. The inoculated medium was mixed well and poured into sterile 10-cmdiameter petri dishes, receiving 15 ml. After setting, 10 cups, each of 8 mm diameter, were cut in the agar medium (Oxoid, Cambridge, England). Twelve milligrams of each extract or fraction or 3 mg of each isolate, accurately weighed, was dissolved in 1 ml dimethyl formamide (DMF). The solutions were inserted in the cups and incubated at 37°C for 24 h. The results of antimicrobial activity are shown in Table 5 and 6.

**Table 5:** Results of antibacterial and antifungal screening of different isolates from *P. guajava* L. leaf

Dry extract/isolate	Inhibition zone (IZ) in mm			
	Bacteria			Fungi
	Gram positive	Gram negative		*C. albicans*
	*S. aureus*	*E. coli*	*P. aeruginosea*	
Aq. ext.	19	16	15	17
16% ethan.	22	17	15	16
50% ethan.	25	17	16	18
95% ethan.	26	17	15	18
P.E.	24	19	—	16
Chloroform.	25	20	—	17
Et. Ac.	16	15	—	20
But.	24	—	—	17
Quercetin	28	17	18	17
Quercetin-3-O-α-L-arabinofuranoside	—	17	—	17
Quercetin-3-O-β-D-arabinopyranoside	—	20	—	18
Ampicillin (10 µg/disk)	35	11	—	—
Clotrimazole (10 mg/ml)	—	—	—	24

Aq. ext., aqueous extract; 16% ethan., ethanolic (16%) extract; 50% ethan., ethanolic (50%) extract; 95% ethan., ethanolic (95%) extract; P.E., petroleum ether fraction of the 50% alcoholic extract; chloroform, chloroform fraction of the 50% alcoholic extract; Et. Ac., ethyl acetate fraction of the 50% alcoholic extract; but., butanol fraction of the 50% alcoholic extract

**Table 6:** MIC of different isolates from *P. guajava* L. leaf

Dry extract/isolate	MIC (µg/ml)		
	*S. aureus*	*E. coli*	*C. albicans*
Aq. ext.	5.25	10.5	5.25
16% ethan.	10.5	10.5	5.25
50% ethan.	5.25	10.5	10.5
95% ethan.	5.25	10.5	5.25
P.E.	7.5	7.5	7.5
Chloroform.	0.78125 (this was least tested concentration)	3.125	6.25
Et. Ac.	2.5	2.5	2.5
But.	5	5	5
Quercetin	1.25	1.25	1.25
Quercetin-3-O-α-L-arabinopyranoside	0.09375	0.1875	0.375
Quercetin-3-O-β-D-arabinopyranoside	0.1875	0.09375	0.1875

Aq. ext., aqueous extract; 16% ethan., ethanolic (16%) extract; 50% ethan., ethanolic (50%) extract; 95% ethan., ethanolic (95%) extract; P.E., petroleum ether fraction of the 50% alcoholic extract; chloroform., chloroform fraction of the 50% alcoholic extract; Et. Ac., ethyl acetate fraction of the 50% alcoholic extract; but, butanol fraction of the 50% alcoholic extract.

## RESULTS AND DISCUSSION

The UV spectra of flavonoid “A” [Table 1] in different shift reagents showed the pattern of 5,7,3’,4’-tetrahydroxy flavonol aglycone, where the presence of free 5- hydroxy and 3’,4’-ortho dihydroxy groups was deduced from NaOMe spectrum, AlCl _3_ and AlCl _3_ /HCl spectra, while the presence of a free 7-hydroxy group was found from the NaOAc spectrum.[35] The electron impact-mass spectrometry (EI-MS) of flavonoid “A” illustrated the presence of molecular ion peak [M]^+^ at *m/z* 302, suggesting the presence of five hydroxyl groups.

^1^H-NMR spectra [Table 2] showed two meta coupled aromatic protons at δ 6.13 and δ 6.4 (d, *J* = 2.1 Hz), assigned for H-6 and H-8, respectively, confirming a 5,7-disubstituted ring A. 3’,4’-disubstituted ring B was deduced by the appearance of three protons at δ 7.71 (d, *J* = 2.15 Hz,), 6.86 (d, *J* = 8.5 Hz) and 7.57 (dd, *J* = 8.5, 2.15 Hz) assigned for H-2’, H-5’ and H-6’, respectively. The ^13^C-NMR spectrum [Table 2] indicated the presence of 15 signals corresponding to 15 carbons. Thorough study of the homonuclear correlation spectroscopy (COSY) and the heteronuclear multiple quantum coherence (HMQC) spectra helped in the full assignment of all protons to their carbon signals. All the spectral data of flavonoid “A” were found to be identical to those reported for quercetin.[31,36] The identification of the flavonoid was further confirmed by direct comparison with reference sample through mixed melting point (m.m.p.) and co-chromatography.

NMR spectra of flavonoids “B and C” [Table 2] showed similar pattern to those of flavonoid “A”, whereas they showed in addition, the appearance of five additional signals in the ^13^C-NMR spectra matching those of arabinose,[36,37] along with the appearance of signals of one sugar moiety in ^1^H-NMR spectra. Therefore, flavonoid “A” was probably the flavonol aglycone and flavonoids “B and C ”were its arabinosides. These data were further supported by the results of the acid hydolysis through comparison of flavonoid “A” and the aglycones resulting from the acid hydrolysis of flavonoids “B and C” with a reference quercetin sample by co-chromatography. Similarly, the sugar moieties were established to be arabinose by comparison with reference sample.

The anomeric proton of flavonoid “B” was observed at δ 5.48 (1H, br s) with its corresponding carbon atom at δ 107.9, while the anomeric proton of flavonoid “C” was observed at δ 5.17 (1H, d, *J* = 6.5 Hz) with its corresponding carbon atom at δ 105, indicating that the arabinose moiety possessed α-configuration inflavonoid “B” and β-configuration in flavonoid “C”. The ring size of the sugar moiety in both flavonoids was deduced from inspection of the chemical shift values for C-1" and C-4" where they appeared at δ 107.9 and 86.4 for flavonoid “B” and at δ 105 and 69.5 for flavonoid “C”, thus revealing the presence of α-arabinofuranoside and β-arabinopyranoside moieties in flavonoids “B” and “C”, respectively.[36,37]

3-O-glycosylation was confirmed from the study of the HMBC spectrum of flavonoid “B” [Table 3], which showed the correlation between the carbon at δ 133.3 (C-3) and the proton at δ 5.48 (H-1"). 2D HMQC, 2D HMBC and 2D COSY allowed the assignment of all protons to their carbons. Also, the UV absorption of band I at 354.5 and 358 nm (371 nm for quercetin aglycone) indicates the absence of free 3-OH.

From the previous discussion, the structure of flavonoids “B” and “C” could be identified as quercetin-3-O-α-L-arabinofuranoside and quercetin-3-O-β-D-arabinopyranoside, respectively. The observed data were found to be similar to those published for these materials.[36,37]

It is worth mentioning that this is the first report for the isolation of quercetin-3-O-β-D-arabinopyranoside from the species *P. guajava* L.

Flavonoids “D” and “E” were identified to be quercetin-3-O-β-D-glucoside and quercetin-3-O-β-D-galactoside through comparison with reference compounds using m.m.p. and co-chromatography using ethyl acetate-formic acid-acetic acid-water (25:2:2:4) as the mobile phase.

The results of antibacterial and antifungal screening [Table 5 and 6] showed that quercetin and its glycosides have strong antibacterial activity against the gram positive *S. aureus,* and the gram negative *E. coli* and *P. aeruginosa.* They also showed antifungal activity against *C. albicans.* It is worth mentioning that the minimum inhibitory concentrations (MIC) of quercetin-3-O-β-D-arabinopyranoside and that of quercetin-3-O-α-L-arabinofuranoside glycosides against the tested organisms were even lower than quercetin itself.

All the extracts showed antibacterial and antifungal activities, whereas the chloroformic fraction of the aqueous-alcoholic extract possessed a strong activity against *S. aureus.*

## CONCLUSION

The above results revealed that quercetin is the main flavonoidal nucleus of guava glycosides. Meanwhile, the antimicrobial testing showed that the extracts and the isolated compounds possess antibacterial and antifungal activities. These findings explain the folkloric use of the extracts as bactericide, in cough, diarrhea, gargles to relieve oral ulcers and inflamed gums wound.
